# Opposing Effects of Nutritional Supply on Bone Health at Different Ages: Based on the National Health and Nutrition Examination Survey Database

**DOI:** 10.3390/nu16060758

**Published:** 2024-03-07

**Authors:** Jieqiong Wei, Yaxi Zhang, Yuehan Yuan, Min Li, Bingfang Zhai, Jihua Chen

**Affiliations:** Department of Nutrition Science and Food Hygiene, Xiangya School of Public Health, Central South University, Changsha 410008, China; wjq123@csu.edu.cn (J.W.); zyxcarol@163.com (Y.Z.); 226912072@csu.edu.cn (Y.Y.); 226912068@csu.edu.cn (M.L.); zbf2000@csu.edu.cn (B.Z.)

**Keywords:** high-fat diet, bone mineral density, NHANES, dietary intake

## Abstract

(1) Background: Nutrients play an essential role in bone health, whether in achieving peak bone mineral density (BMD) or maintaining bone health. This study explores the relationship between nutrient supply and femoral bone health at different ages. (2) Methods: A total of 5603 participants meeting the inclusion and exclusion criteria were included in this study using the National Health and Nutrition Examination Survey (NHANES) database from 2005 to 2010, 2013 to 2014, and 2017 to 2018. Femoral bone mineral density and bone status were dependent variables, and dietary nutrient intake and nutrient intake status were independent variables. The relationship between dietary nutrient intake and bone mineral density was explored, and the importance of nutrients affecting bone status was analyzed through a neural network model. At the same time, we investigated the relationship between nutrient intake and bone status. (3) Results: The peak of age and femoral bone mineral density appeared at 20 years old in our study. After grouping by age, logistic regression analysis showed that before 20 years old, without adjusting other variables, high-fat diet was more likely to have normal bone mass than appropriate fat diet (OR: 4.173, 95%CI: 1.007–17.289). After adjusting for all demographic factors, niacin intake (OR: 1.062, 95%CI: 1.019–1.108) was beneficial for normal bone mass, while vitamin B_6_ intake (OR: 0.627, 95%CI: 0.408–0.965) was not. After 20 years old, after adjusting for carbohydrate, protein, vitamin B_6_, niacin, dietary fat, vitamin B_2_, and vitamin B_12_, vitamin B_2_ intake (OR: 1.153, 95%CI: 1.04–1.278) was beneficial for normal bone mass, while vitamin B_6_ intake (OR: 0.842, 95%CI: 0.726–0.976) was not. After adjusting for all confounding factors, vitamin B_2_ intake (OR: 1.288, 95%CI: 1.102–1.506) was beneficial for normal bone mass. In addition, we found that even if there was no statistical significance, the effects of high-fat diet on bone mass were different at different ages. (4) Conclusions: By conducting an in-depth analysis of the NHANES database, this study reveals that dietary factors exert divergent effects on bone health across different age groups, implying the necessity of implementing tailored dietary strategies to maintain optimal bone health at distinct life stages.

## 1. Introduction

Osteoporosis (OP) is a metabolic bone disease characterized by reduced bone mass and the deterioration of bone tissue microstructure, leading to increased susceptibility to fractures due to compromised bone strength [1]. Due to its asymptomatic nature, it is often referred to as a “silent disease” [2,3]. Moreover, osteoporotic fractures are frequently comminuted fractures that exhibit poor stability in internal fixation treatment, thereby predisposing individuals to delayed or non-union of fractures. Consequently, there is an urgent need for the exploration of more effective preventive and therapeutic measures. Current research has identified that OP progression primarily correlates with peak bone mineral density and the subsequent continuous loss of bone mass [4,5], emphasizing the significance of maximizing peak bone mass and delaying the onset of age-related decline in skeletal health for OP prevention and treatment [6]. Given that long bone fractures, particularly femoral fractures, contribute significantly to the disease burden associated with osteoporosis, identifying factors influencing femoral peak bone mineral density and implementing appropriate interventions are crucial for effective OP prevention and management.

The formation of peak bone mass is influenced by genetics, environment, and nutrition. Among these factors, genetics and environment are immutable and account for 44–88% of bone mineral density formation. Conversely, the remaining variability in bone mineral density is closely associated with behavioral factors such as nutrition [7,8]. Previous studies on nutritional factors have primarily focused on the relationship between individual nutrient characteristics and bone mineral density [9]. However, research attention has been limited to adults rather than children and adolescents who undergo significant growth and development [6]. Considering that their nutritional needs differ greatly from those of adults [6], it is crucial to identify which dietary factors are most beneficial for improving bone health across different age groups. In analyzing complex high-dimensional data, machine learning techniques like artificial neural networks offer distinct advantages over traditional statistical methods as they can better identify key features and facilitate data analysis.

Inspired by the artificial model of biological neurons [8], artificial neural networks have garnered significant attention from researchers as a nonlinear approach for investigating the relationship between input and output information [10]. Serving as an effective method for general classification, clustering, and prediction, the advantage of utilizing neural networks lies in their ability to adapt to diverse datasets without making any assumptions about the underlying model structure [11,12]. In recent years, neural networks have been applied in various domains related to bone health, including detecting and classifying bone lesions [13] and assessing bone age [11]. However, there is currently limited utilization of neural network models in exploring factors influencing bone health.

Therefore, the objective of this study is to investigate the impact of dietary factors on bone mineral density and bone mass across different age groups within a large population using neural network analysis and correlation analysis based on the NHANES database. The utilization of a neural network model enables the assessment of the significance of dietary factors at various life stages in relation to bone health, while also examining specific dietary nutrients that exert the greatest influence on bone status. These findings aim to provide innovative theoretical foundations for regulating and preventing issues pertaining to bone health.

## 2. Materials and Methods

### 2.1. Data Sources

The National Health and Nutrition Examination Survey (NHANES) is a cross-sectional population survey administered by the US Centers for Disease Control and Prevention (CDC) to assess the health and nutritional status of adults and children in the United States. This study has been approved by the National Center for Health Statistics (NCHS) Institutional Review Board, and informed consent was obtained from all participants (https://www.cdc.gov/nchs/nhanes/index.htm, accessed on 6 March 2024). Since 1999, the National Health and Nutrition Examination Survey (NHANES) has conducted biennial surveys with a nationally representative sample, consisting mainly of interviews and physical examinations. The interviews include demographic, socioeconomic, and diet- and health-related questions, while the physical examination includes physiological measurements and laboratory tests.

**Figure 1:**
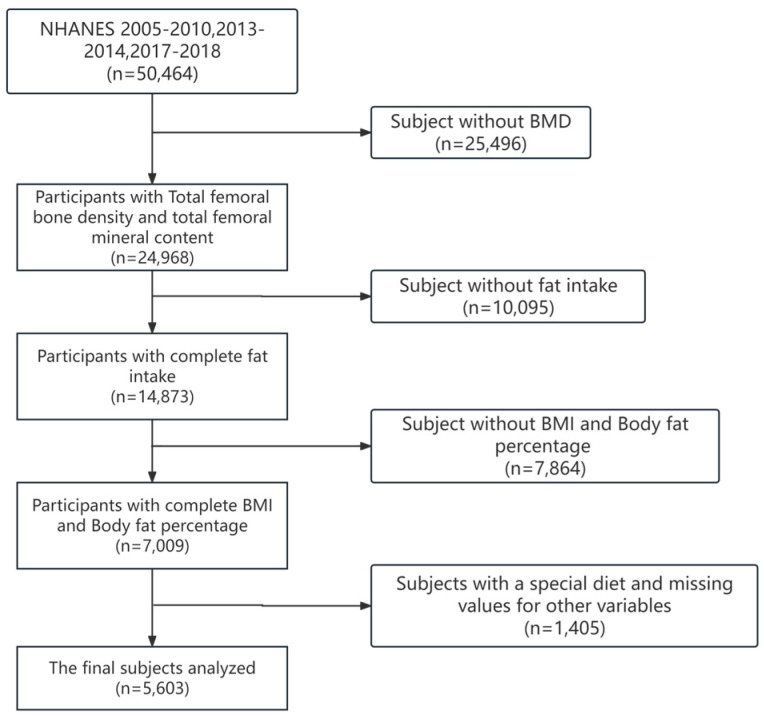
Flowchart for selecting research participants.

Demographic data from three portions (2015–2010; 2013–2014; and 2017–2018) were combined with dietary survey data for analysis. During the periods of 2015–2010, 2013–2014, and 2017–2018, there were 50,464 participants in the NHANES. We excluded participants who did not complete the survey or had total femoral BMD values, dietary data were missing or unreliable, and had special diets. Then, we matched the participants’ demographic and sociological information with the dietary survey data according to each participant’s code. Finally, we included 5603 participants (3005 males and 2598 females) in the analysis (Figure 1).

### 2.2. Evaluation of Dietary Data

Nutrient and food data were obtained from the total nutrition profiles, which contained the total nutrients of all the foods and beverages consumed by individuals provided in the diet recall. The intake status of protein, carbohydrate, and total fat was calculated based on nutrient intake, nutrient capacity coefficient, and total energy (National Center for Health Statistics). According to the WHO recommendations for the supply ratio of the three major capacity nutrients, the percentage of carbohydrate supply in total energy was defined as low-carbohydrate diet, higher than 75% as high-carbohydrate diet, and between 55% and 75% as appropriate carbohydrate diet; the recommended supply ratio of protein was 10–15%, which was defined as low-protein diet, appropriate protein diet, and high-protein diet; and the supply ratio of fat was defined as appropriate fat diet at less than 30%, and higher than 30% was defined as high-fat diet.

### 2.3. Assessment of Bone Mineral Density

We selected femoral BMD as the dependent variable in the analysis. Qualified survey participants aged 8 years and older underwent DXA scans to obtain femoral BMD, which can be obtained from the DXXFEM dataset. Femoral BMD was measured by dual energy X-ray absorptiometry (DXA) using a Hologic densitometer (Hologic, Inc., Bedford, MA, USA) and software Apex 3.2. Femoral BMD values were collected and standardized by professionals.

### 2.4. Covariates

The CAPI system was used to obtain demographic characteristics. These included gender, age, race (Mexican American, other Hispanic, non-Hispanic white, non-Hispanic black, and other races), education level (below 9th grade, 9–11th grade, high school graduation/GED or equivalent, college or above), marital status (widowed, divorced or separated, unmarried, and cohabiting), number of family members, and annual household income.

### 2.5. Statistical Analysis

Continuous variables with normal distribution, such as bone mineral density and dietary nutrient data, were expressed as mean ± SD, and demographic data were expressed as categorical variables with n (%). A *t*-test was used to evaluate the difference between normal and reduced bone mass subjects. In addition, the chi-square test was used to assess categorical demographic differences. Multivariate logistic regression was used to analyze the association between dietary factors and bone status while controlling for other covariates. The odds ratio (OR) and 95% confidence interval (95%CI) were used to reflect the direct relationship between dietary factors and femoral bone status. The restricted cubic spline model was used to analyze the relationship between age and femoral bone mineral density. Then, a two-piecewise linear regression model was constructed to calculate the turning point. The neural network model was used to analyze the reasons for the early peak age of femoral bone mineral density. Firstly, we preprocessed and prepared the dietary data and femoral bone mineral density in the NHANES database. In order to better build the evaluation model, we converted the bone mineral density data into bone status according to age, race, and gender using the following calculation formula: T-value = (patient’s bone mineral density value − the average bone mineral density of non-patient adults) ÷ the standard deviation of normal adult bone mineral density. Since our T-value conversion is only based on the data of this study and cannot absolutely define osteoporosis, we divided the bone status into normal bone mass and reduced bone mass, with T-value ≥ −1 for normal bone mass and T-value < −1 for reduced bone mass, which were used as dependent variables. We used the neural net package of R to construct an artificial neural network model, with various nutrient intake as the input variable and bone mass status as the output variable. We set the number of nodes and layers of the hidden layer and set appropriate activation and loss functions. To ensure the accuracy of the model, we used 70% of the dataset as the training set and 30% as the test set and used the prediction accuracy of the test set to evaluate the fitting effect of the model. The weight and deviation parameters of the model were used to explain the degree of influence of different factors on bone mineral density. Through the output results of the neural network model, we can quantitatively evaluate the degree of influence of each variable on bone status. Statistical correlation analysis was conducted using SPSS 25.0, and the construction of an artificial neural network model and data visualization were conducted using R 4.3.0. The OR (odds ratio) value represents the influence of the independent variables on the dependent variables; *p* < 0.05 was considered statistically significant.

## 3. Results

### 3.1. Characteristics of Subjects

The characteristics of the subjects of different ages are shown in Table 1, including the sociodemographic data, physical examination data, and dietary data of the participants selected from the NHANES (2005–2010, 2013–2014, and 2017–2018). In this study, we finally included 5603 subjects, with 2105 (37.57%) aged less than or equal to 20 years old and 3498 (62.43%) aged older than 20 years old. Among the subjects aged less than or equal to 20 years old, there were 1100 males and 1005 females. Among the subjects aged older than 20 years old, there were 1905 males and 1593 females. Except for gender (*p* = 0.109), there were statistically significant differences in sociodemographic data between the ≤20 years old and >20 years old groups (*p* < 0.001). The total femoral bone mineral density of the ≤20 years old group was 0.91 ± 0.18 g/cm^2^, while that of the >20 years old group was 0.96 ± 0.15 g/cm^2^. At the same time, the BMI of the ≤20 years old group was 21.59 ± 4.68 kg/m^2^, while that of the >20 years old group was 26.97 ± 5.20 kg/m^2^. There were significant differences in bone mineral density and body mass index between the different age groups (*p* < 0.05).

With the exception of energy intake (*p* = 0.198), thiamine (*p* = 0.448), vitamin C (*p* = 0.268), iron (*p* = 0.093), zinc (*p* = 0.683), and sodium (*p* = 0.957), there were no statistically significant differences observed in the majority of macronutrient and micronutrient intake across different age groups, as indicated by *p*-values greater than 0.05 (Table 2).

### 3.2. Correlation between Age and Bone Mineral Density

**Figure 2:**
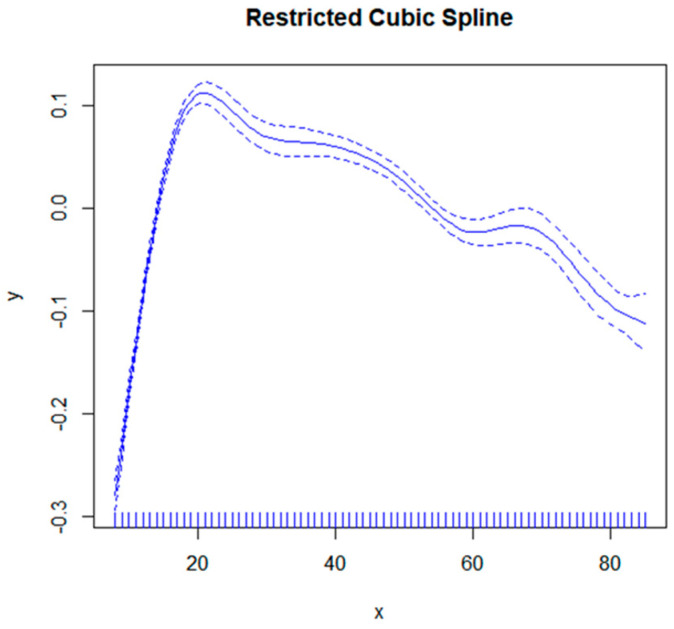
The correlation between age and femoral bone density. The solid blue line represents a smoothed curve fit between variables. The blue dashed band represents the 95% confidence interval for the fit.

The correlation between age and total femoral bone mineral density (Figure 2) exhibits a nonlinear pattern, with an inverse relationship observed before and after the peak at 20.16 years old. Specifically, there is a positive association between age and femoral bone mineral density when ≤20 years old, while a negative association is evident beyond the age of 20 (Table 3).

### 3.3. Machine Learning Using Neural Network Models

**Figure 3:**
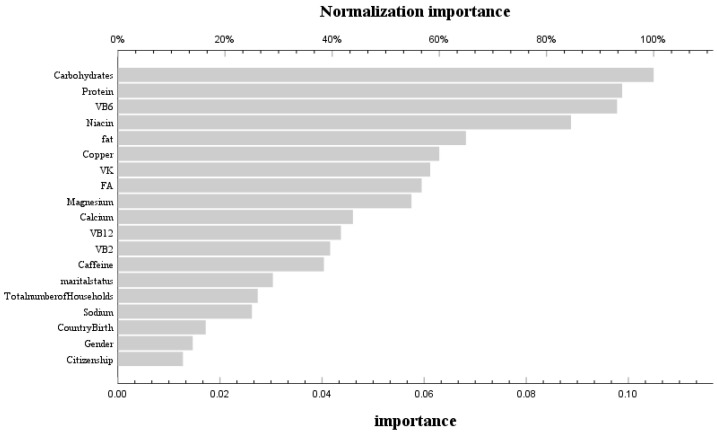
Use of neural network models to score the importance and normal importance of selected variables before 20 years old.

**Figure 4:**
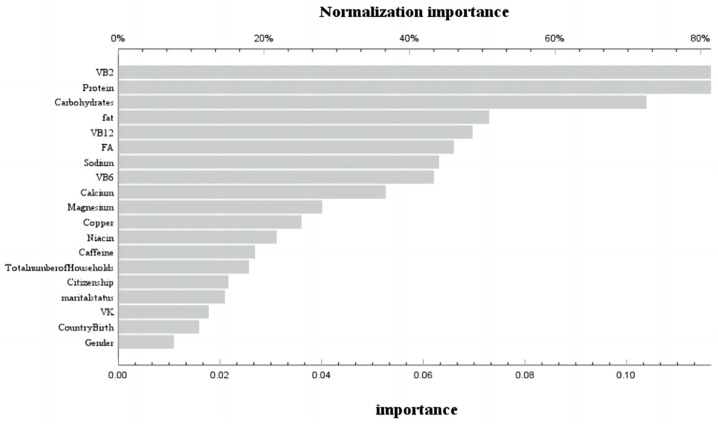
Use of neural network models to score the importance and normal importance of selected variables after 20 years old.

During the phase of model development and validation, we employed machine learning techniques and artificial neural network models to ascertain the relative significance of dietary variables associated with bone mass status at different stages of life. These variables encompassed macronutrients (carbohydrates, protein, fat, and cholesterol) as well as micronutrients (vitamin A, vitamin E, total folate, vitamin B_12_, vitamin C, vitamin K, calcium, phosphorus, magnesium, selenium, caffeine, thiamine, niacin, and vitamin B6). The receiver operating characteristic (ROC) curves for the neural network models across various age groups are depicted in Figure S1. The corresponding area under the curve (AUC) values were found to be 0.601 and 0.571, respectively, indicating a satisfactory fit of the models. In the age group ≤ 20 years, the training set achieved a prediction accuracy of 85%, while the prediction set achieved an accuracy of 86.1%. For the age group > 20 years, the training set had a prediction accuracy of 84.5%, and the prediction set had an accuracy of 85.2%. By assessing the contribution of variables in our neural network model, we identified that before reaching 20 years old, carbohydrates, protein, VB_6_, niacin, and dietary fat were found to be the five most influential factors affecting femoral bone mineral density (Figure 3). Conversely, after surpassing 20 years old, VB_2_, dietary protein, carbohydrates, dietary fat, and VB_12_ emerged as the five most significant variables influencing femoral bone mineral density (Figure 4).

After age-based grouping, logistic regression analysis was conducted to examine the association between the aforementioned dietary factors and femoral bone mineral density. The results indicated that individuals in the high-fat diet group were more likely to have normal bone mineral density before 20 years old (OR: 4.173, 95%CI: 1.007–17.289) without adjusting for other variables. However, after adjusting for all demographic factors, it was found that niacin intake had a beneficial effect on normal bone mineral density (OR: 1.062, 95%CI: 1.019–1.108), while vitamin B_6_ intake did not show any significant benefit (OR: 0.627, 95%CI: 0.408–0.965) (Table 4 and Table S2). After the age of 20, following adjustments for carbohydrate, protein, vitamin B_6_, niacin, dietary fat, vitamin B_2_, and vitamin B_12_ intake levels, it was observed that higher vitamin B_2_ intake (OR: 1.153; 95%CI: 1.04–1.278) had a positive association with normal bone mineral density. Conversely, no significant benefit on normal bone mineral density was found for vitamin B_6_ intake (OR: 0.842; 95%CI: 0.726–0.976). Furthermore, after accounting for all confounding factors in the analysis model, increased vitamin B_2_ intake remained significantly beneficial for maintaining normal bone mineral density (OR: 1.288; 95%CI: 1.102–1.506) (Table 4, Tables S1 and S2). Additionally, although not statistically significant across different age groups, the impact of a high-fat diet on bone mineral density varied.

## 4. Discussion

In our cross-sectional study, we identified that the age of 20 serves as a turning point in the relationship between age and femoral bone density. Before 20 years old, femoral bone mineral density showed a significant positive correlation with age, while after 20 years old, it showed a negative correlation. The peak of femoral bone mineral density was reached at 20 years old. At the same time, we found through an artificial neural network model for machine learning that in dietary factors, carbohydrates, protein, vitamin B_6_, niacin, and dietary fat were the five most important variables affecting femoral bone mineral density before 20 years old. In contrast, after 20 years old, it was vitamin B_2_, dietary protein, carbohydrates, dietary fat, and vitamin B_12_. In the case of no adjustment for other variables, a high-fat diet was a protective factor for normal bone before 20 years old. After adjusting carbohydrates, protein, vitamin B_6_, niacin, dietary fat, vitamin B_2_, and vitamin B_12_, it was found that vitamin B_2_ was a protective factor for normal bone after 20 years old, while vitamin B_6_ was a risk factor for normal bone. After adjusting for all confounding factors, we found that vitamin B_6_ was a risk factor for normal bone mass before the age of 20, while niacin was a protective factor. After passing the age of 20, vitamin B_2_ was a protective factor for normal bone mass. Furthermore, we found that the effects of a high-fat diet on femoral bone mineral density showed opposite trends in different age groups. Compared with a normal diet, a high-fat diet was beneficial for bone mineral density before the age of 20, but it became a risk factor for bone mineral density after the age of 20. This result suggests that a high-fat diet has different effects in different age groups.

Previous studies investigating the changes in femur bone mineral density have focused more on adults or postmenopausal women [14,15]. In our study, all adolescents and adults before the age of 18 were included in the analysis to observe the changes in femoral bone mineral density throughout the life cycle. Our results showed that peak bone mineral density was reached in our population at the age of 20, which is consistent with the results of a longitudinal study on bone mineral density conducted by Claudie Berger in Canada [16]. The results showed that the peak bone mineral density of the femoral neck was designated at the ages of 16 to 19 years old in women and the age of 17 years old in men. The bone mineral density of the femoral neck began to decline after 19 years old, which is consistent with most studies conducted in Western countries. In any case, a cross-sectional study conducted in South Korea to evaluate the relationship between hypogonadism and bone mineral density found that the bone mineral density of patients with isolated hypogonadotropic hypogonadism decreased as early as 25 years old, which is significantly earlier than the control group [17]. A population study conducted in Taiyuan found that the peak bone mineral density of the femoral neck occurred at the ages of 31–35 years old in women and at about 25 years old in men [18]. Based on the above studies, the peak bone mass age of Eastern populations is later than that of Western populations. Previous studies have found that the factors impacting the peak bone mass of both groups include genetic variation, environmental factors, and dietary nutrition factors. Among them, genetic and environmental factors, as unchangeable factors, can explain 44% to 88% of BMD [19,20]. But as controllable and variable factors, behavioral factors such as nutrition have the greatest impact on the residual variability of bone mass [21].

Existing studies on the impact of dietary patterns on bone mass have found that the consumption of fruits and vegetables is a protective factor for high bone mineral density. The precise mechanisms underlying this phenomenon remain elusive; however, the high concentration of vitamins and minerals found in fruits and vegetables is proposed as a potential explanatory factor [22]. For nutrients traditionally associated with bone health, such as vitamin D and calcium, studies have found that although bones need them to maintain stability, consuming more vitamin D and calcium is not beneficial for bone health in all cases [23]. In addition to the above two factors, the effects of other nutrients on bone mineral density are still inconclusive, and the extent to which each factor affects bone mineral density is unknown.

We constructed an artificial neural network model with socio-economic factors and dietary nutrient intake as independent variables and bone status as dependent variables. Further analysis of the independent variables revealed that carbohydrates, dietary protein, VB_6_, niacin, and dietary fat were the five most important variables affecting femoral bone mineral density before the age of 20, while VB_2_, dietary protein, carbohydrates, dietary fat, and VB_12_ were the five most important variables affecting femoral bone mineral density after the age of 20 [24]. Correlation analysis found that VB_6_ was a risk factor for normal bone before 20 years old, while niacin was a protective factor. After 20 years of age, VB_2_ was a protective factor for normal bone. We found that vitamin B_2_ was a protective factor for normal bone after 20 years of age, which is consistent with the results of Abrahamsen et al. Adult women with the lowest vitamin B_2_ intake had the lowest femoral neck bone mineral density and the highest risk of fracture [25]. In vitro, experiments have shown that B_2_ and its photoproducts can reduce the RANKL (receptor activator of nuclear factor-κB ligand)/OPG (osteoprotegerin) ratio by enhancing the expression of OPG in pre-osteoblast MC3T3-E1 cells [26]. At the same time, we found that VB_6_ before 20 years old is a risk factor for normal bone, while most current studies on adults have found that lower B_6_ levels are associated with higher risks of bone loss and hip fractures [27]. Conversely, some studies have shown that the effect of vitamin B_6_ on bone health may change due to age, gender, and disease status, which may be the reason for different results [27]. Niacin after 20 years old is a protective factor, but most studies have found that niacin intake in the diet of premenopausal women is significantly positively correlated with BMD. So far, few studies have explored the effect of niacin on bone health before the age of 20 years. Two early studies from the same laboratory found that when young [28] and old [29] chickens were supplemented with different doses of niacin, there was no significant difference in bone mineral content in chickens, which requires further research. At the same time, although there was no statistical difference, we found a negative correlation between vitamin B_12_ and bone status before the age of 20. This is similar to the findings of Ann et al., in that vitamin B_12_ deficiency was associated with low BMD, increased risk of fractures, and osteoporosis. This may be related to the risk of vitamin B_12_ deficiency [30], which leads to the increased stimulation of osteoclastogenesis and the inhibition of osteoblasts.

Our study found that according to the classification and correlation analysis of the three major nutrient function ratios, low-carbohydrate diet and high-protein diet were risk factors for bone status after 20 years old. This is consistent with the research results that low-carbohydrate diet is more beneficial to prevent bone loss, and high-carbohydrate intake is associated with a decrease in distal radial BMD [31]. Researchers believe that this may be closely related to the form of carbohydrates. Current evidence suggests that diets high in refined sugar may affect bone growth and strength, with sucrose-fed rats having lower femur strength [32], while high-glucose interventions are detrimental to osteoblast proliferation and differentiation [33], and high-fructose diets also reduce calcium ion transport in animals [34]. While most studies have found that cellulose can increase bone mass, Kim found that calcium ion absorption increased in adults and menopausal women after using inulin interventions [35]. Only one prospective study has shown a negative correlation between dietary protein and bone [36]. This is consistent with our findings, where the researchers suggested that this negative correlation may be due to low calcium intake in children. This was supported by the report by Vatanparast et al., where the positive effect of dietary protein on bone mass was most significant in those who consumed sufficient calcium (>1000 mg/d) [37]. More studies have shown that long-term protein intake positively predicts whole-body BMC. Most prospective and cross-sectional studies support evidence of a positive correlation between protein intake and bone [38,39]. In studies on the effects of three major energy nutrients on bone mineral density, researchers have differing views on the effects of fat intake, or high-fat diet (HFD), on bone mineral density [40,41]. HFD has a complex effect on bone health. Early studies have treated weight gain as a protective factor for bone health, while HFD causes weight gain [42]. A cohort study of bone mineral density and body weight in elderly men and women conducted at Framingham found that body weight and weight change had a strong effect on bone mineral density, which was often beneficial [43].

On the other hand, most recent studies have found that short-term HFD can induce bone loss [44,45], while long-term HFD-exposed mice had reduced bone mass, which may be mainly due to the increase in adipocytes by affecting the bone marrow microenvironment [46]. Although healthy weight gain can benefit bone health by increasing the load-bearing capacity, these benefits may be lost in the case of HFD-induced weight gain [47]. A survey of a population’s bone mineral density and lifestyle habits showed that fatty diets were the most important risk factor for early bone loss in women, except for genetics. Most studies support the idea that an HFD can regulate the differentiation of bone marrow mesenchymal stem cells and affect bone health. Compared with the osteogenic differentiation of bone marrow mesenchymal stem cells, consuming a high amount of fat leads to the differentiation of bone marrow mesenchymal stem cells into adipocytes, which accumulate in the bone marrow cavity and form bone marrow adipose tissue (BMAT) [48]. Studies have found that a decrease in bone mass is often accompanied by an increase in bone marrow adipose tissue. Bone marrow obesity caused by an HFD will expand adipose tissue and lead to chronic low-grade systemic inflammation [45]. The accumulation of ectopic adipocytes in the bone marrow cavity may damage bone regeneration and lead to bone fat imbalance, leading to osteoporosis [46]. In contrast, our study found that a high-fat diet has opposite effects in different age groups. It is a protective factor before 20 years of age, but it becomes a harmful factor after 20 years of age. We believe that this may be the reason why the peak of bone mineral density in Western populations occurs earlier than that in Eastern populations. High-fat diet has a similar effect to “credit card” on bone mineral density, which overdraws bone mineral density at an earlier age. Although there was no statistical difference, we found that vitamin B12 was negatively correlated with bone status before the age of 20. This trend of a negative impact of nutrient intake due to age may provide a basis for better dietary recommendations to improve bone health in the future.

Though it is important to note that other factors should also be considered in the use of the neural network model, the accuracy and stability of the model should be further verified and adjusted [47]. Firstly, age is an important variable that is usually closely related to bone health. In the neural network model we built, although we used age as a stratification factor, we used 20 years old as the dividing line, which focused more on the factors of bone loss in youth. Similarly, gender is also an important variable that may affect bone status. However, we found no statistical correlation between gender and bone status in the preliminary correlation analysis results, which goes against most people’s research [20,48]. It is generally believed that women have a lower peak bone mass than men throughout their lives, and their bone loss rate after menopause is faster than men’s. Studies have shown that women have a higher prevalence of osteoporotic fractures than men. At the same time, women may be more susceptible to the adverse effects of high-fat diets, leading to a decline in bone status. Through further research and validation, we can gain a deeper understanding of the contribution of each variable to bone status.

Compared to previously published articles, this study possesses several advantages. Firstly, the utilization of NHANES data ensures high-quality data through meticulous and continuous quality control measures. Our study employed a substantial sample size of 5403 participants and determined that the peak age for population bone mineral density occurs at 20 years old. Secondly, we applied machine learning techniques utilizing artificial neural network algorithms to assess the contribution of each dietary variable to bone mineral density in different age groups, thereby investigating the factors influencing early peak bone mineral density.

However, our study has certain limitations. Firstly, the demographic and dietary data were self-reported, which may introduce measurement errors and recall bias. Secondly, our study design was cross-sectional in nature, necessitating prospective studies to elucidate the potential associations between dietary factors, age, and bone mineral density. Additionally, due to the unavailability of recorded vitamin D intake in the National Health and Nutrition Examination Survey (NHANES), it could not be included in our analysis.

## 5. Conclusions

By conducting an in-depth analysis of the NHANES database, this study reveals that dietary factors exert divergent effects on bone health across different age groups, implying the necessity of implementing tailored dietary strategies to maintain optimal bone health at distinct life stages.

## Figures and Tables

**Table 1:** Demographic characteristics of the study subjects (*n* = 5603).

Characteristics	Total Number of Subjects (*n* = 5603)		Statistic	*p* Value
	≤20	>20		
Gender			2.565	0.109
Male	1100	1905		
Female	1005	1593		
Country of Birth			175.729	<0.001
Mexican Americans	1856	2577		
Other Hispanic	175	739		
Non-Hispanic white	74	182		
Citizenship			22.335	<0.001
Born or naturalized citizens	1897	3006		
Non-US citizens	208	487		
Others	0	5		
Marital status			2640.290	<0.001
Married	11	2050		
Widowhood	1	217		
Divorce	0	430		
Separation	8	108		
Never married	1087	458		
Living with a partner	33	234		
other	965	1		
Total number of households			1007.355	<0.001
1	21	509		
2	150	1028		
3	348	708		
4	572	562		
5	505	377		
6	265	160		
>6	244	154		
Annual household income			383.878	<0.001
less than USD 20,000	449	629		
USD 20,000 to 44,999	636	1059		
USD 45,000 to 74,999	474	756		
More than USD 75,000	526	527		
others	20	527		
Total femoral density(g/cm^2^)	0.91 ± 0.18	0.96 ± 0.15	94.554	<0.001

**Table 2:** Dietary intake of study subjects (*n* = 5603).

Characteristics	Total Number of Subjects (*n* = 5603)		Statistic	*p* Value
	≤20	>20		
Energy (kcal)	2262.71 ± 1082.29	2221.44 ± 1055.19	−1.286	0.198
Protein (g)	79.16 ± 44.09	84.90 ± 47.08	−4.843	<0.01
Carbohydrates (g)	296.38 ± 147.61	266.60 ± 131.92	−7.885	<0.01
Total fat (g)	85.52 ± 46.55	83.56 ± 48.21	−2.336	0.019
Fat energy supply	769.66 ± 418.91	752.04 ± 433.92	−2.336	0.019
The proportion of fat energy supply	0.34 ± 0.08	0.33 ± 0.09	−1.53	<0.01
Cholesterol (mg)	256.61 ± 212.22	307.22 ± 251.91	−7.341	<0.01
Vitamin E	6.43 ± 4.80	7.95 ± 5.86	−11.018	<0.01
Total folic acid (μg)	398.83 ± 300.08	409.99 ± 248.63	−4.312	<0.01
Vitamin B_12_ (μg)	5.38 ± 5.01	5.50 ± 8.62	−3.571	<0.01
Vitamin C (mg)	93.72 ± 107.25	87.86 ± 102.63	−1.108	0.268
Vitamin K (μg)	63.04 ± 80.98	106.32 ± 152.39	−16.597	<0.01
Calcium (mg)	984.85 ± 639.68	924.24 ± 594.45	−3.308	<0.01
Phosphorus (mg)	1303.05 ± 695.55	1376.44 ± 705.68	−4.446	<0.01
Magnesium (mg)	245.21 ± 130.73	307.18 ± 157.02	−17.139	<0.01
Iron (mg)	16.21 ± 9.85	15.63 ± 9.15	−1.681	0.093
Zinc (mg)	11.88 ± 7.71	12.26 ± 12.86	−0.408	0.683
Selenium (μg)	105.01 ± 60.74	116.62 ± 72.25	−6.146	<0.01
Caffeine (mg)	39.99 ± 72.38	176.02 ± 221.89	−33.095	<0.01
Vitamin B_2_ (mg)	2.15 ± 1.31	2.23 ± 1.33	−2.425	0.015
Vitamin B_1_(mg)	1.69 ± 1.01	1.67 ± 1.01	−0.758	0.448
Niacin (mg)	24.34 ± 15.14	26.11 ± 15.96	−4.999	0.957
Vitamin B_6_ (mg)	1.86 ± 1.28	2.10 ± 1.40	−7.146	<0.01

**Table 3:** Correlation analysis between age and femoral bone density at different stages (*n* = 5603).

Age	Pearson’s Correlation	*p* Value
≤20 years	0.658	<0.001
>20 years	-0.307	<0.001

**Table 4:** Logistic regression analysis of dietary factors and bone status.

	Model 1			Model 2			Model 3		
	b	*p* Value	OR	b	*p* Value	OR	b	*p* Value	OR
≤20 years									
Vitamin B_2_	0.021	0.65	1.021	−0.098	0.163	0.906	−0.349	0.075	0.705
Vitamin B_6_	0.064	0.15	1.066	0.001	0.996	1.001	−0.466	0.034	0.627
Niacin	0.006	0.093	1.006	0.012	0.181	1.012	0.061	0.004	1.062
Vitamin B_12_	0.008	0.495	1.008	0	0.995	1	0	0.983	1
Carbohydrates stage									
Appropriate		0.251			0.422			0.818	
Low	−0.186	0.152	0.83	0.233	0.346	1.263	0.074	0.772	1.077
High	−0.304	0.201	0.738	0.077	0.761	1.08	−0.219	0.578	0.803
Protein stage									
Appropriate		0.188			0.221			0.082	
Low	−0.289	0.125	0.749	0.193	0.174	1.212	−0.567	0.079	0.567
High	−0.183	0.169	0.833	−0.068	0.764	0.935	−0.32	0.251	0.726
HFD stage									
Appropriate									
High	1.429	0.049	4.173	1.388	0.057	4.007	1.142	0.286	3.133
>20 years									
Vitamin B_2_	0.024	0.478	1.025	0.142	0.007	1.153	0.253	0.002	1.288
Vitamin B_6_	−0.058	0.104	0.943	−0.172	0.022	0.842	−0.125	0.168	0.883
Niacin	−0.003	0.288	0.997	0.004	0.584	1.004	0.003	0.765	1.003
Vitamin B_12_	−0.003	0.653	0.997	−0.003	0.689	0.997	−0.005	0.696	0.995
Carbohydrates stage									
Appropriate		0.031			0.057			0.068	
Low	−0.123	0.283	0.884	−0.117	0.327	0.889	−0.166	0.258	0.847
High	0.292	0.109	1.339	0.291	0.117	1.338	0.333	0.089	1.396
Protein stage									
Appropriate		0.077			0.356			0.529	
Low	0.266	0.085	1.304	0.214	0.177	1.239	0.183	0.303	1.2
High	−0.08	0.425	0.923	−0.01	0.924	0.99	−0.068	0.622	0.934
HFD stage									
Appropriate			1						
High	−0.035	0.897	0.966	−0.051	0.852	0.95	−0.047	0.878	1.048

Model 1 does not adjust any variables; in model 2, 7 variables were adjusted. Model 3 adjusts all variables such as age, gender, race, marital status, income poverty ratio, and education level.

## Data Availability

All NHANES data for this study are publicly available and can be found here: https://wwwn.cdc.gov/nchs/nhanes (accessed on 15 January 2024).

## Supplementary Materials

The following supporting information can be downloaded at: https://www.mdpi.com/article/10.3390/nu16060758/s1, Figure S1: ROC curves of neural network models at different age stages; Table S1: Logistic regression analysis of bone status in research subjects of different age groups (model 2); Table S2: Logistic regression analysis of bone status in research subjects of different age groups (model 3).
