# Supplementary material for: Opposing Effects of Nutritional Supply on Bone Health at Different Ages: Based on the National Health and Nutrition Examination Survey Database
Source: Nutrients. 2024 Mar 7;16(6):758. doi: 10.3390/nu16060758 (PMC10974100; doi:10.3390/nu16060758)
Supplement: Supplementary file 1 [file nutrients-16-00758-s001.zip › nutrients-2880950-supplementary.pdf]

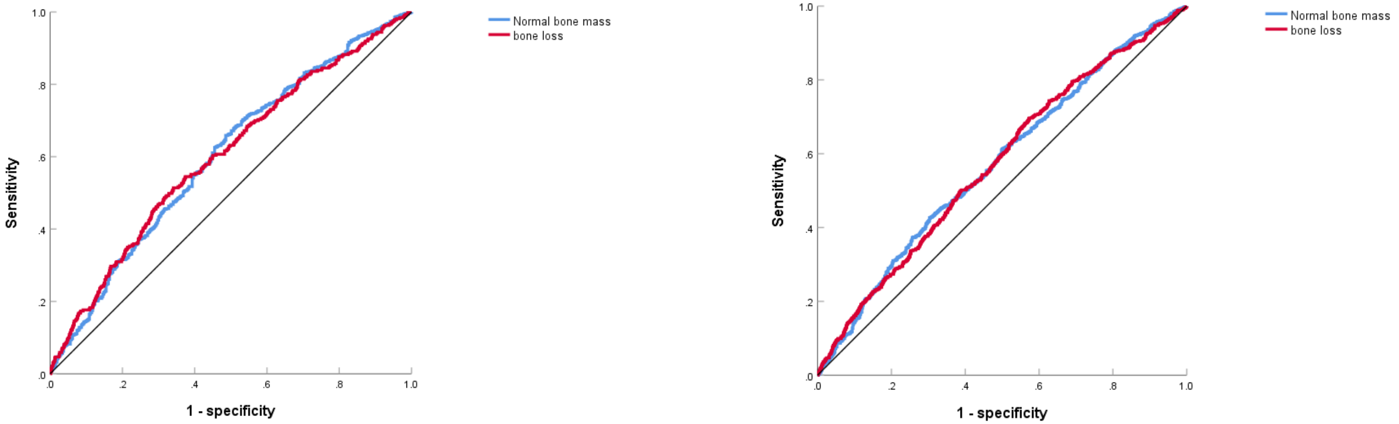

Supplementary Figure S1: ROC curves of neural network models at different age stages.

Opposing Effects of Nutritional Supply on Bone Health at Different Ages: Based on the NHANES Database

Supplementary Table S1. Logistic regression analysis of bone status in research subjects of different age groups (modal 2).

	b	Sb	Wald χ2	p-Value	OR	95% CI	
						lowest	highest
≤20 years							
vitamin B_2	−0.098	0.071	1.943	0.163	0.906	0.789	1.041
vitamin B_6	0.012	0.009	1.788	0.181	1.012	0.995	1.029
Niacin	0.001	0.105	0	0.996	1.001	0.815	1.228
vitamin B_{12}	0	0.015	0	0.995	1	0.971	1.03
Carbohydrates stage							
Appropriate			1.724	0.422			
low	0.233	0.247	0.89	0.346	1.263	0.778	2.05
high	0.077	0.252	0.092	0.761	1.08	0.659	1.769
Protein stage							
Appropriate			3.017	0.221			
low	0.193	0.142	1.851	0.174	1.212	0.919	1.6
high	−0.068	0.225	0.09	0.764	0.935	0.601	1.453
HFD stage							
Appropriate							
high	1.388	0.728	3.632	0.057	4.007	0.961	16.699
constant	−3.367	0.781	18.572	0	0.034		
<20 years							
vitamin B_2	0.142	0.053	7.327	0.007	1.153	1.04	1.278
vitamin B_6	0.004	0.007	0.299	0.584	1.004	0.991	1.017
Niacin	−0.172	0.075	5.236	0.022	0.842	0.726	0.976

vitamin B_{12}	−0.003	0.006	0.16	0.689	0.997	0.985	1.01
Carbohydrates stage							
Appropriate			5.718	0.057			
low	−0.117	0.12	0.96	0.327	0.889	0.703	1.124
high	0.291	0.186	2.456	0.117	1.338	0.93	1.926
Protein stage							
Appropriate			2.066	0.356			
low	0.214	0.159	1.821	0.177	1.239	0.908	1.691
high	−0.01	0.104	0.009	0.924	0.99	0.807	1.214
HFD stage							
Appropriate							
high	−0.051	0.272	0.035	0.852	0.95	0.558	1.62
constant	−1.722	0.138	156.168	0	0.179		

Supplementary Table S2. Logistic regression analysis of bone status in research subjects of different age groups (modal 3).

	b	Sb	Wald χ2	p-Value	OR	95% CI	
						lowest	highest
≤20 years							
home PIR	−0.087	0.059	2.179	0.14	0.916	0.816	1.029
Gender							
male	0	0.182	0	0.998	1	0.701	1.428
female					1		
Country of Birth							
Mexican Americans	0.457	0.622	0.54	0.462	1.58	0.467	5.349
Other Hispanic	−1.395	0.72	3.754	0.053	0.248	0.06	1.016
Non-Hispanic white					1		
Citizenship							
Born or naturalized citizens	−1.392	0.832	2.795	0.095	0.249	0.049	1.271
Non-US citizens					1		
marital status							
married	−16.849	1	284.069	0	4.81×10^{-8}	6.79×10^{-9}	3.42×10^{-7}
Widowhood	−0.308	7908.451	0	1	0.735	0	3.72×10^{-7}
divorce	−16.886	1.054	256.449	0	4.64×10^{-8}	5.88×10^{-9}	3.67×10^{-7}
separation	−16.648	0.559	888.32	0	5.89×10^{-8}	1.97×10^{-8}	1.76×10^{-7}
Never married	−16.363	0	.	.	7.83×10^{-8}	7.83×10^{-8}	7.83×10^{-8}
Living with a partner	−1.231	7908.451	0	1	0.292	0	7.23×10^{-8}

others					1		
Total number of households							
1	0.705	0.951	0.55	0.458	2.023	0.728	4.255
2	0.565	0.45	1.575	0.209	1.76	0.91	4.059
3	0.653	0.381	2.932	0.087	1.922	0.879	3.384
4	0.545	0.344	2.515	0.113	1.725	0.698	2.477
5	0.274	0.323	0.72	0.396	1.315	0.781	3.261
6	0.467	0.365	1.641	0.2	1.596	.	.
>6					1		
high-fat diet							
Appropriate	−0.573	0.271	4.48	0.034	0.564	0.331	0.958
high					1		
Total Fat(g)	−0.013	0.01	1.708	0.191	0.987	0.968	1.007
Energy(kcal)	0	0.001	0.097	0.756	1	0.998	1.001
Carbohydrates(g)	0.003	0.003	0.781	0.377	1.003	0.997	1.008
Protein(g)	−0.005	0.006	0.709	0.4	0.995	0.984	1.007
Dietary fiber(g)	−0.031	0.015	4.068	0.044	0.97	0.941	0.999
Total Saturated fat acids(g)	0.033	0.019	3.191	0.074	1.034	0.997	1.072
Cholesterol(mg)	0.001	0.001	4.001	0.045	1.001	1	1.003
Vitamin C (mg)	0	0.001	0.133	0.716	1	0.999	
Vitamin K(μg)	0.001	0.001	0.636	0.425	1.001	0.998	1.002
Calcium(mg)	0	0	0.051	0.821	1	0.999	1.004
Phosphorus(mg)	0	0	0.114	0.736	1	0.999	1.001

Magnesium(mg)	0.001	0.002	0.082	0.775	1.001	0.997	1.001
Iron(mg)	−0.019	0.014	1.758	0.185	0.982	0.955	1.004
Zinc(mg)	−0.008	0.017	0.23	0.631	0.992	0.958	1.009
Copper(mg)	0.524	0.335	2.446	0.118	1.689	0.876	1.026
Sodium (mg)	0	0	0.307	0.58	1	1	3.255
vitamin E	−0.013	0.03	0.201	0.654	0.987	0.93	1.047
vitamin A	−0.001	0	2.142	0.143	0.999	0.999	1
vitamin B_1	0.121	0.224	0.293	0.588	1.129	0.728	1.751
vitamin B_2	−0.349	0.196	3.171	0.075	0.705	0.48	1.036
Niacin	0.061	0.021	8.097	0.004	1.062	1.019	1.108
vitamin B_6	−0.466	0.22	4.507	0.034	0.627	0.408	0.965
FA	−0.001	0.001	3.66	0.056	0.999	0.997	1
vitamin B_{12}	0	0.023	0	0.983	1	0.955	1.047
vitamin C	−0.001	0.001	0.587	0.444	0.999	0.997	1.001
vitamin K	0.001	0.001	0.2	0.655	1.001	0.998	1.003
vitamin E	−0.013	0.03	0.201	0.654	0.987	0.93	1.047
vitamin A	−0.001	0	2.142	0.143	0.999	0.999	1

<20 years							
home PIR	0.028	0.043	0.444	0.505	1.029	0.946	1
Gender							
male	0.1	0.137	0.531	0.466	1.105	0.845	1
female							
Country of Birth							
Mexican Americans	0.457	0.622	0.54	0.462	1.58	0.467	.
Other Hispanic	−1.395	0.72	3.754	0.053	0.248	0.06	5.349
Non-Hispanic white							
Citizenship							
Born or naturalized citizens	−1.392	0.832	2.795	0.095	0.249	0.049	.
Non-US citizens							
marital status							
married	−16.849	1	284.069	0	4.81×10^{-8}	6.79×10^{-9}	.
Widowhood	−0.308	7908.451	0	1	0.735	0	3.42×10^{-7}
divorce	−16.886	1.054	256.449	0	4.64×10^{-8}	5.88×10^{-9}	.C
separation	−16.648	0.559	888.32	0	5.89×10^{-8}	1.97×10^{-8}	3.67×10^{-7}
Never married	−16.363	0	.	.	7.83×10^{-8}	7.83×10^{-8}	1.76×10^{-7}
Living with a partner							
others							
Total number of households							
1	0.748	0.813	0.847	0.357	2.113	0.43	.

2	0.26	0.373	0.484	0.487	1.297	0.624	10.394
3	0.339	0.323	1.1	0.294	1.404	0.745	2.695
4	0.316	0.31	1.035	0.309	1.372	0.746	2.645
5	0.017	0.305	0.003	0.955	1.017	0.56	2.52
6	0.275	0.351	0.613	0.434	1.317	0.661	1.848
>6					1		
high-fat diet							
Appropriate	−0.573	0.271	4.48	0.034	0.564	0.331	1.566
high					1		
Total Fat(g)	−0.013	0.01	1.708	0.191	0.987	0.968	1.029
Energy(kcal)	0	0.001	0.097	0.756	1	0.998	1.007
Carbohydrates(g)	0.003	0.003	0.781	0.377	1.003	0.997	1.001
Protein(g)	−0.005	0.006	0.709	0.4	0.995	0.984	1.008
Dietary fiber(g)	−0.031	0.015	4.068	0.044	0.97	0.941	1.007
Total Saturated fat acids(g)	−0.998	2.427	0.169	0.681	0.369	0.003	.
Cholesterol(mg)	0.033	0.019	3.191	0.074	1.034	0.997	42.932
Vitamin C (mg)	0.001	0.001	4.001	0.045	1.001	1	1.072
Vitamin K(μg)	0.001	0.001	0.636	0.425	1.001	0.998	1.002
Calcium(mg)	0	0	0.051	0.821	1	0.999	1.004
Phosphorus(mg)	0	0	0.114	0.736	1	0.999	1.001
Magnesium(mg)	0.001	0.002	0.082	0.775	1.001	0.997	1.001
Iron(mg)	−0.019	0.014	1.758	0.185	0.982	0.955	1.004
Zinc(mg)	−0.008	0.017	0.23	0.631	0.992	0.958	1.009
Copper(mg)	0.524	0.335	2.446	0.118	1.689	0.876	1.026

Sodium (mg)	0	0	0.307	0.58	1	1	3.255
vitamin E	−0.009	0.013	0.445	0.505	0.991	0.965	1.018
vitamin A	0	0	0.188	0.665	1	1	1
vitamin B_1	0.134	0.083	2.635	0.105	1.143	0.973	1.344
vitamin B_2	0.253	0.08	10.077	0.002	1.288	1.102	1.506
Niacin	0.003	0.01	0.089	0.765	1.003	0.984	1.022
vitamin B_6	−0.125	0.091	1.897	0.168	0.883	0.739	1.054
FA	−0.001	0	4.546	0.033	0.999	0.998	1
vitamin B_{12}	−0.005	0.013	0.153	0.696	0.995	0.97	1.021
vitamin C	−0.001	0.001	1.403	0.236	0.999	0.998	1.001
vitamin K	0	0	0.649	0.421	1	0.999	1.001
